# Quantifying the Impact of Deployments of Autonomous Vehicles and Intelligent Roads on Road Safety in China: A Country-Level Modeling Study

**DOI:** 10.3390/ijerph20054069

**Published:** 2023-02-24

**Authors:** Hong Tan, Fuquan Zhao, Haokun Song, Zongwei Liu

**Affiliations:** 1 State Key Laboratory of Automotive Safety and Energy, Tsinghua University, Beijing 100084, China; 2 Tsinghua Automotive Strategy Research Institute, Tsinghua University, Beijing 100084, China

**Keywords:** autonomous vehicle, intelligent road, vehicle to vehicle, road safety, collision reduction, crash-related economic cost

## Abstract

Approximately 1.35 million people lose their lives due to road traffic collisions worldwide per year. However, the variation of road safety depending on the deployment of Autonomous Vehicles (AV), Intelligent Roads (IR), and Vehicle-to-Vehicle technology (V2V) is largely unknown. In this analysis, a bottom-up analytical framework was developed to evaluate the safety benefits of avoiding road injuries and reducing crash-related economic costs from the deployment of AVs, IRs, and V2Vs in China in 26 deployment scenarios from 2020 to 2050. The results indicate that compared with only deploying AVs, increasing the availability of IRs and V2V while reducing the deployment of fully AVs can achieve larger safety benefits in China. Increasing the deployment of V2V while reducing the deployment of IRs can sometimes achieve similar safety benefits. The deployment of AVs, IRs, and V2V plays different roles in achieving safety benefits. The large-scale deployment of AVs is the foundation of reducing traffic collisions; the construction of IRs would determine the upper limit of reducing traffic collisions, and the readiness of connected vehicles would influence the pace of reducing traffic collisions, which should be designed in a coordinated manner. Only six synergetic scenarios with full equipment of V2V can meet the SDG 3.6 target for reducing casualties by 50% in 2030 compared to 2020. In general, our results highlight the importance and the potential of the deployment of AVs, IRs, and V2V to reduce road fatalities and injuries. To achieve greater and faster safety benefits, the government should prioritize to the deployment of IRs and V2V. The framework developed in this study can provide practical support for decision-makers to design strategies and policies on the deployment of AVs and IRs, which can also be applied in other countries.

## 1. Introduction

Every year, 1.35 million people are killed and 50 million are injured globally in road traffic accidents [1]. Road traffic crashes are a leading cause of death and serious injury. In addition, their economic and social consequences are massive. Fatal and nonfatal crash injuries are estimated to cost the world economy approximately $1.8 trillion dollars from 2015–2030 [2]. Recognizing its public health and international development priority, road safety is a key target under Sustainable Development Goal 3.6, with a 50% reduction in road traffic fatalities called for by 2020 [3]. In China, there were 61,703 and 65,225 people killed in traffic collisions in 2020 and 2010 [4] The decrease in traffic fatalities was only 5.4% between 2010 and 2020 in China. Similar to China, there were 35,766 and 30,296 fatal crashes in the United States in 2020 and 2010 [5], with an increase of 18% between 2010 and 2020. Some goals have been set to encourage countries and social agendas to pay more attention and effort to road safety. The UN General Assembly proclaims the Second Decade of Action for Road Safety 2021–2030, with a goal to reduce deaths and injuries by 50% by 2030 [6], which is an adjustment to the target date for Sustainable Development Goal 3.6. First implemented in Sweden in the 1990s, Vision Zero is a strategy to achieve a transportation system with no fatalities or serious injuries involving road traffic, which has been adopted by European Union, Columbus, Boston, and many other cities [7]

A huge challenge for achieving SDG3.6 goals and Vision Zero is the fact that China’s vehicle stocks will keep rapid growth for a long time in the future. China’s vehicle ownership has experienced rapid growth with an increase of 250% in the past decade. Such explosive growth keeps the number of traffic accidents consistently high [8], although the number of traffic fatalities per thousand vehicles has decreased by 73%. The saturation level of vehicle ownership per 1000 people is estimated to be approximately 376 [9] while the figure is 193 in 2020. China is now the world’s largest auto market, with 26.3 million and approximately 28 million vehicles sold in 2021 and 2022. Increasing the use of seatbelts, optimization for side impacts, and improvements to vehicle front-end design were estimated to result in 12.1%, 6.3%, and 6.0% fewer traffic deaths [10], which is not enough to support the achievement of ambitious SDG 3.6 goals. In the foreseeable future, effective new technologies should be introduced to reduce traffic deaths and injuries by 50%.

Much of the political and social agenda is focused on discovering the next innovative vehicle technology to improve road safety. AV technology is regarded as a high-tech solution to transform the current approach to automotive safety from injury reduction post-collision to complete collision prevention [11]. Achieving SDG 3.6 goal and Vision Zero on road safety is not a task for one industry or one technological field. AVs offer an exceptional opportunity to reduce road injuries. However, AVs are not a magical universal solution, and the arsenal should include a wide range of policies and technologies combined with Intelligent roads [12,13,14,15,16] and Vehicle-to-Vehicle [17,18,19].

In this paper, the safety benefits of reducing road injuries and saving in crash-related economic costs from the deployment of AVs, IRs, and V2Vs in China in 26 out of 48 scenarios from 2015 to 2050 were estimated. The aim was to assess potential road safety benefits from individual and combined strategies applied to AVs, IRs, and V2V and to illustrate the variation of these potentials by the proportion of the different levels of autonomous vehicles in vehicle stocks, the proportion of intelligent roads, and proportion of vehicle stocks with V2V. In total, 6 out of 26 synergetic strategies were identified to meet the SDG 3.6 target for reducing injuries by 50% compared to 2020, and 6 out of 26 scenarios were identified to be close to achieving the goal. We discuss and derive insights for policymakers on ways to appropriately address the deployment of AVs, IRs, and V2Vs from the perspective of road safety.

The developed framework, which applies to any country provided the availability of road safety and transportation data, may provide practical support for decision-makers to design strategies and policies on autonomous vehicles, intelligent roads, and V2V by quantifying the impact on reducing road crashes and saving crash-related economic costs.

The next section describes the method, required data and processing details of the study. Following that, the result of annual fatalities related to road crashes, cumulative reductions in road fatalities, and cumulative savings in crash-related economic costs in different deployment scenarios are provided. The subsequent section shows the discussion and policy implications and conclusions. The final section provides the conclusion.

## 2. Methods and Materials

In this section, the analytical framework and the description of scenarios are provided first. Following that, the details of the data including vehicle sales, mileage of roads, baseline fatalities per billion kilometers traveled, collision avoidance effectiveness and unit economic cost of road collisions in China are presented.

### 2.1. Framework

**Figure 1:**
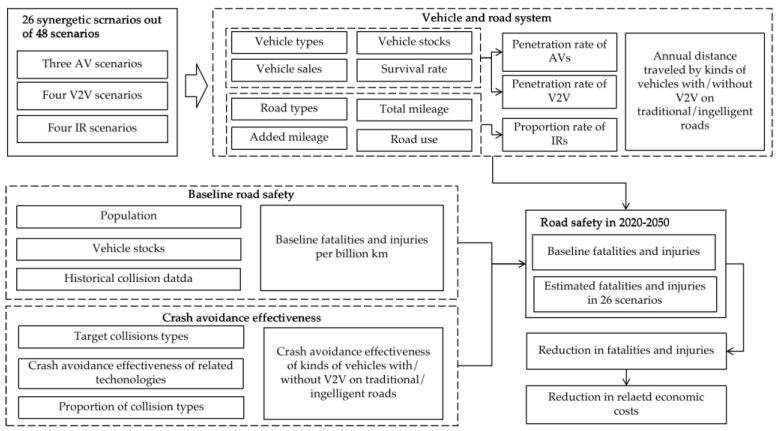
The analytical framework used in this study.

An analytical framework was developed to evaluate the number of fatalities and injuries that would be avoided, as well as the crash-related economic costs that would be reduced in China in the 26 out of 48 synergetic scenarios of deployment of AVs, IRs, and V2V, as shown in Figure 1. The other 16 scenarios that have no practical significance are not considered in this study, including 16 scenarios that do not deploy AVs but deploy V2V and IRs, and 6 scenarios that do not deploy V2V but deploy IRs. A bottom-up method was developed to evaluate the road traffic fatality in China based on vehicle stocks, annual distance traveled, fatalities and injuries per billion kilometers traveled, the proportion of the different levels of autonomous vehicles in vehicle stocks, proportion of distance traveled on the intelligent road, proportion of vehicle stocks with V2V, crash avoidance effectiveness of different levels of autonomous vehicles driving on the traditional or intelligent road with V2V working or without V2V working, as shown in Equation (1). The reduction in crash-related economic costs could be evaluated based on the number of crashes with different degrees of injury in the baseline, the number of crashes in synergetic scenarios, and the unit economic cost of crashes in China, as shown in Equation (2).

(1) $${\mathrm{Fata}}_{y,s}=\sum_{v=0}^{3}(VS_{v,y}\times AVKT\times FPT_{y,base}\times \sum_{r=0}^{1}\left(PTR_{r,y}\times \left(PVV_{y}{}^{2}\times \left(1-Eff_{v,r,1}\right)+\left(1-PVV_{y}{}^{2}\right)\times \left(1-Eff_{v,r,0}\right)\right)\right)$$

(2) $${\mathrm{ECS}}_{y,s}=\overset{3}{\underset{d=0}{\sum }}\left(\left({\mathrm{Crash}}_{y,d,\mathrm{baseline}}-{\mathrm{Crash}}_{y,d,s}\right)\times {\mathrm{UEC}}_{d}\right),$$

where

${\mathrm{Fata}}_{y,s}$ are the road fatalities in the scenario s in China in the year y;
$VS_{v,y}$ are the vehicle stocks of v type of vehicles in the year y;
v = 0, 1, 2, 3 represent traditional vehicles, primary AVs, partially AVs and fully Avs, respectively;
AVKT is the average annual distance traveled in a lifespan of the vehicle in China;
$FPT_{y,base}$ represents baseline traffic fatalities per billion km traveled in China in year y;
r  = 0.1 represent the traditional road and intelligent road, respectively;
$PTR_{r,y}$ is the proportion of distance traveled on the intelligent road (r = 1) or the traditional road (r = 0) in the year y based on the development of intelligent road;
$PVV_{y}$ is the proportion of vehicle stocks with V2V;
$Eff_{v,r,vv}$ is the crash avoidance effectiveness of v type of vehicles driving on the traditional (r = 0)/intelligent (r = 1) road with V2V working (vv = 1) or without V2V working (vv = 0);
vv = 0.1 represent the with V2V working and without V2V working, respectively;
${\mathrm{ECS}}_{y,s}$ are the total economic costs saved by the AV, IRs and V2V in China in the year y in the s scenario;
d = 0, 1, 2, 3 represent PDO, minor injuries, severe injuries, and fatalities, respectively;
${\mathrm{Crash}}_{y,d,s}$ is the number of crashes with degree of injury d in the s scenario in the year y;
${\mathrm{Crash}}_{y,d,\mathrm{baseline}}$ is the number of crashes with degree of injury d in the baseline in the year y;
${\mathrm{UEC}}_{d}$ is the unit economic cost of road collisions resulted in degree of injury d.

### 2.2. Description of Scenarios

The 48 intelligent transportation system scenarios (3 × 4 × 4) are developed, defined by three scenarios describing the deployment of autonomous vehicles, four scenarios describing the deployment of intelligent road mileage, and four scenarios describing the equipping of Vehicle-to-Vehicle technology (Table 1). The scenarios incorporate different approaches to achieve the visionary but ambitious UN Sustainable Development Goal (SDG) 3.6 to halve the number of road traffic deaths and injuries as soon as possible. The national efforts and development in the three technical fields of autonomous vehicles, intelligent roads, and V2V would have different impacts on fatalities and injuries in traffic collisions. Vehicle-side approaches are represented in the AV market share scenarios and V2V equipment scenarios. The degree of automation and market penetration of autonomous vehicles are used to define AV scenarios. The vehicle types equipped with V2V technology are selected in V2V scenarios. Road-side approaches are represented in intelligent road scenarios. The speed of intelligent road construction and the types of roads covered are set in IR scenarios. The scenarios are not optimized to achieve a specific road collision reduction target.

### 2.3. Data

#### 2.3.1. Vehicle Sales and Stocks in China

The method is widely used and applied to calculate the vehicle stock based on the historical vehicle stock, vehicle sales, and survival rates in this article [22,23], as shown in Equation (3). The penetration rate of types of AVs in vehicle sales is obtained from the technology roadmap for intelligent and connected vehicles 2.0 in China and scenario definition for AVs [20,24]. The penetration rate of V2V is obtained from the definition of the scenarios for V2V.

(3) $${\mathrm{VS}}_{v,y}=\overset{y}{\underset{j=y-l}{\sum }}{\mathrm{Sales}}_{j}\times {\mathrm{PR}}_{v,y}\times {\mathrm{SR}}_{y-j},$$

where ${\mathrm{VS}}_{v,y}$ is the vehicle stocks of v type of AVs in the target year y;

v is the type of vehicles, traditional vehicles (v = 0), primary AVs (v = 1), partially AVs (v = 2) and fully AVs (v = 3);
l is the lifespan of the vehicle;
${\mathrm{Sales}}_{j}$ is the number of vehicle sales in year j;
${\mathrm{PR}}_{v,y}$ is the penetration rate of v type of AV in the vehicle sales in year y;
${\mathrm{SR}}_{y-j}$ is the survival rate of the vehicle in the ${\left(y-j\right)}_{\mathrm{th}}$ year (%).

#### 2.3.2. Coverage Rate and Mileage of Intelligent Roads

The historical total mileage of different road types in China was obtained from the National Bureau of Statistics. The mileage growth of various highways was obtained from the National Highway Network Plan 2022–2035 and official statistics [25,26]. The mileage growth of urban roads was obtained by the “National Urban Infrastructure Construction Plan of the Fourteenth Five Year Plan” [27]. The coverage ratio of intelligent roads in various roads was obtained by “The White Paper on C-V2X Industrialization Path and Schedule” issued by China ITS Industry Alliance and the scenario definitions for IRs [21]. Therefore, the proportion of intelligent roads on the types of roads could be calculated in scenarios of IRs.

The proportion of distance traveled on intelligent roads depends on the proportion of intelligent roads on the types of roads and the distribution of vehicle distance traveled on various types of roads, as shown in Equation (4). The proportion of intelligent roads on the types of roads is obtained from the scenario definitions. The distribution of driving distance on various types of roads in lifespan is obtained from the historical data.

(4) $$PTR_{1,y}=\overset{7}{\underset{t=1}{\sum }}\left(DDR_{t}\times RIR_{t,y}\right),$$

where

${\mathrm{PTR}}_{1,y}$ is the proportion of distance traveled on the intelligent road in the year y;
t = 1, 2, … 8 represents road types including urban expressways, urban ordinary roads, expressways, Class I highways, Class II highways, Class III highways, Class IV highways and substandard roads;
${\mathrm{DDR}}_{t}$ is the distribution of driving distance traveled on the road type t;
${\mathrm{RIR}}_{t,y}$ is the proportion of intelligent roads on the roads type t in China in the year y.

#### 2.3.3. Baseline Fatalities Per Billion Kilometers Traveled

Smeed’s formula [28] and Brosos’s formula [29] were widely used to calculate and predict the country-level fatalities and injuries per 1000 registered vehicles or per billion km traveled based on the population [30], vehicle stock, and historical road collision data [4], as shown in Equations (5) and (6). After comparing the accuracy of the prediction results, the Smeed model was selected for this research. The national annual average Vehicle Kilometers Traveled (VKT) in the vehicle lifespan is obtained from the historical research of our research group [22,31]. The historical road crash data are obtained from “The Annual Report on Road Traffic Accidents” [4]. The predicted population in China is reported by the United Nations [30].

(5) $$FPT_{y,base}=e^{a1}\times {\left(\frac{VS_{y}}{Pop_{y}}\right)}^{-b1}÷AVKT\times 10^{9},$$

(6) $$FPT_{y,base}=a_{2}\times e^{-b_{2}\times \frac{VS_{y}}{Pop_{y}}}÷AVKT\times 10^{9},$$

where FPTy represents baseline traffic fatalities per billion km traveled in China in year y;

${\mathrm{Pop}}_{y}$ is the population of China in year y;
AVKT is the average annual distance traveled in the vehicle lifespan in China;
${\mathrm{VS}}_{y}$ are the vehicle stocks in the target year y;
$a_{1}$, $b_{1}$, $a_{2}$ and $b_{2}$ are parameters of the formulas obtained by fitting historical data.

According to the statistics of traffic crashes in the past few years, the number of serious injuries, minor injuries, and PDO collisions in road traffic collisions in China is approximately 4 times, 42 times, and 135 times the number of fatalities, respectively [4]. Therefore, traffic injuries and PDO crashes could be predicted and calculated based on the result of fatalities.

#### 2.3.4. Comprehensive Collision Avoidance Effectiveness

A crash avoidance effectiveness evaluation model was developed for estimating the safety effectiveness of autonomous vehicles enabled with different sensor combinations based on multiple variables of 14 different “atomic” sensing technologies on the vehicle side and road side, 52 safety functions, and 14 accident types [32], which has been published. Atomic technologies refer to the safety-related basic hardware of autonomous vehicles, which means perception-related hardware in this paper, such as cameras, millimeter wave radar, LiDAR, etc. A total of 52 safety functions including 26 safety functions that rely on vehicle-side sensors and 26 connected safety functions that depend on roadside sensors are covered, such as Automatic Emergency Braking (AEB) and Connected AEB, Lane Change Assistance (LCA) and Connected LCA [33]. Proportions of 14 types of collisions (e.g., sideswipe collision, rear-end collision, and head-on collision) in China’s road traffic collisions are considered in the model. The map of comprehensive collision avoidance effectiveness of all vehicle and road side “atomic” technology combinations are provided in the published paper [32]. The comprehensive collision avoidance effectiveness of different levels of autonomous vehicles driving on the traditional or intelligent road used in this study are shown in Table 2. For partially AV, the collision avoidance effectiveness of the partially AV itself is estimated to be 55.6%, and the effectiveness rises to 74.7% when V2V also works. The effectiveness of the partially AV driving on the IR is estimated to be 94.6%. The effectiveness increases to 96.7% when the partially AV is driving on the IR and the surrounding vehicle is also equipped with V2V. For fully AV, the collision avoidance effectiveness values are estimated to be 89.1%, 92.3%, 98.3%, and 98.8%, respectively.

#### 2.3.5. Unit Economic Cost of Road Collisions in China

The annual comprehensive economic losses of road collisions in the United States, United Kingdom, Sweden, Germany, Australia and India have been estimated [34,35]. A comprehensive model was proposed in our previously published research to evaluate the economic loss of road traffic crashes in China based on the data obtained from official Chinese agencies [36]. The economic losses caused by road collisions include medical costs, lost productivity, travel delay time cost, legal and court costs, insurance costs, and property damage. The total economic losses from traffic collisions in China were estimated to be USD 72.6 billion in 2017, which was equivalent to 0.60% of the GDP of China [36]. The more serious the injury, the higher the unit’s economic loss. The unit economic loss of a PDO collision, minor injured collision, severe injured collision, and fatal collision was estimated to be USD 1670, USD 2995, USD 128,195, and USD 471,192 in 2017, respectively. More details on the economic losses of road crashes can be found in our previously published paper [36].

## 3. Results

In this section, the details of the deployment of AVs, IRs and V2V are introduced first. The following parts show annual fatalities in 26 synergetic scenarios. Then, deployments for achieving SDG target 3.6 in 2030 are identified. After that, the cumulative reductions in road fatalities and the cumulative savings of crash-related economic costs in 26 scenarios are given.

### 3.1. Deployment of AVs, IRs, and V2V

**Figure 2:**
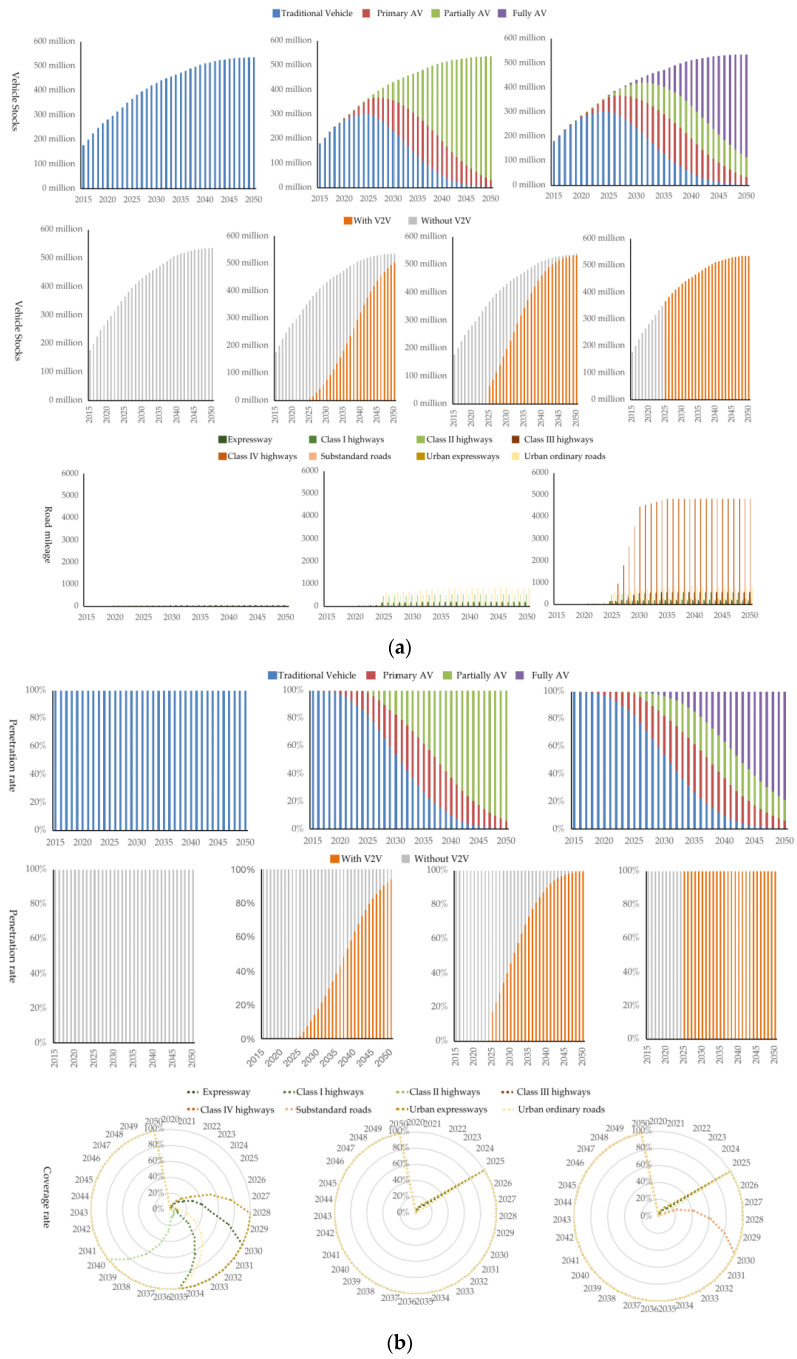
Deployment of AVs, IRs, and V2V. (**a**) Vehicle stocks of AVs and V2V; road mileage of IRs in China from 2015 to 2050; (**b**) Proportion of AVs and V2V in the vehicle stocks; the proportion of IRs in different road types from 2015 to 2050.

The vehicle market in China will maintain a momentum of robust growth. Vehicle sales will gradually increase from 28.4 million in 2022 to 34.9 million in 2030 and then remain at approximately 30 million per year. The vehicle stocks will also maintain rapid growth, from 317.7 million in 2022, which exceeded the mark of 300 million for the first time, to 506 million in 2039, which pass the mark of 500 million for the first time. Autonomous vehicles have become the main focus of the automobile industry in China. The government, automobile manufacturers, technology enterprises, and other stakeholders are investing a lot of resources to promote the rapid development of AVs. The proportion of primary AVs in vehicle sales reached approximately 20% in 2021 and increased to 32.4% in 2022 [24]. According to the plan in the AV roadmap, the proportion of primary AV and partially AV in car sales would reach 50% in 2025, and fully AVs will begin to enter the market. By 2035, 100% of vehicle sales will be AVs of different levels [20]. According to our assessment, the proportions of primary AV, partially AV, and fully AVs in the vehicle stocks in 2030 are estimated to be 28.3%, 14.1%, and 3.4%, respectively, while they will become 27.1%, 26.3%, and 36.6% in 2040, respectively, as shown in Figure 2.

Regarding V2V, the proportion of vehicles with V2V in the vehicle stocks will gradually increase in RGAV-APV2V, RGAV-HV2V, and RGAV-FV2V scenarios, and the difference between scenarios will gradually become narrow. The proportion of vehicles with V2V in the vehicle stocks will vary greatly in 2025, estimated to be 1.5%, 17.5%, and 100%, respectively, while the proportion will reach 17.5, 45.8%, and 100%, respectively, in 2030. In 2050, the proportion of V2V among different scenarios will be very close, estimated to be 93.9%, 99.5%, and 100%, respectively.

China’s highways and urban roads have experienced flourishing development, with a total highway mileage of 5281 thousand kilometers and a total urban road mileage of 517 thousand by 2021. From the perspective of road types, the total mileage of the urban expressways, urban ordinary roads, expressways, Class I highways and Class II highways, Class III highways, Class IV highways, and substandard roads in China are 169, 128, 426, 467, 3871, 2190, 24 and 493 thousand kilometers in 2021, respectively [25]. The mileage of all kinds of the road will keep growing. The proportion of IRs in the kinds of road types each year is estimated, as shown in the Figure. By 2030, under the APIR, GRIR, and FIRIR scenarios, the mileage covered by intelligent roads is estimated to be 489, 1380, and 6548 thousand kilometers, respectively, with huge differences. In 2050, the total mileage covered by IRs in both APIR and GRIR is estimated to be 1747 thousand kilometers, while the total mileage covered by IRs in FIR will reach 7425 thousand kilometers, as shown in Figure 1. The mileage covered by IRs in the FIR scenario is 4.3 times that of APIR and GRIR, because the mileage of Class III highways, Class IV highways, and substandard roads is pretty large.

### 3.2. Annual Road Fatalities in China

If AVs or IRs are not introduced to transportation, road traffic injury will still be severe in the baseline scenario. The historical number of road fatalities was 61,703 in 2020 [4]. The estimated baseline road fatalities will rise slightly to 63,972 in 2030, and then decline slightly to 60,876 and 58,421 in 2040 and 2050, respectively. The road fatalities per billion kilometers decreased by 36% from 0.16 in 2020 to 0.10 in 2030, and are estimated to be 0.09 and 0.08 in 2040 and 2050. Vehicle ownership increased by 58% from 273.4 million in 2020 to 432.1 million in 2030, and is estimated to be 512 million and 536 million in 2040 and 2050. As a result, the annual road fatalities in the future will increase slightly and then stay at a high position compared with the fatalities in 2020, although the number of traffic fatalities per thousand vehicles will decrease.

**Figure 3:**
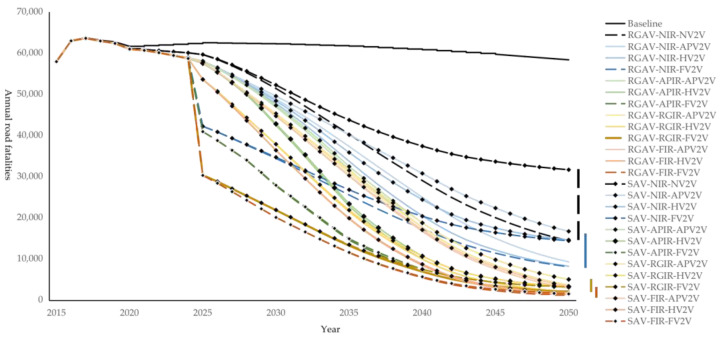
Annual road fatalities for 26 scenarios. Annual road fatalities are summarized in each AV scenario (RGAV, SAV), IR scenario (NIR, APIR, GRIR, and FIR), and V2V scenario (NV2V, APV2V, HV2V, and FV2V) combination, which appear in 27 lines. The symbol represents each AV scenario, with the color representing the IR scenario and the depth of color representing the V2V scenario.

Annual fatalities related to road crashes declined in 24 synergetic scenarios and two only-AV scenarios, as shown in Figure 3. The foundation of the decline is the development of autonomous vehicles. RGAV-NIR-NV2V reduces road fatalities by 5%, 17%, 52%, and 75%, while SAV-NIR-NV2V is estimated to reduce road fatalities by 5%, 16%, 38%, 46% in 2025, 2030, 2040, and 2050. If the country only promotes autonomous vehicles without paying attention to deploying V2V and IR, on the one hand, the speed of reducing traffic fatalities will be very slow because it requires a long time for AV to penetrate into the vehicle stock; on the other hand, the potential of reducing traffic fatalities is not very high; even by 2050, the annual road fatalities will not be reduced to a very low level.

The extent of the decline is largely affected by IR coverage. The more types of roads covered by intelligent roads, the more road fatalities can be reduced in the future. In 2050, FIR can reduce more road fatalities than APIR and NIR. For example, the estimated road fatalities in 2050 for RGAV-NIR-FV2V, RGAV-APIR-FV2V, and RGAV-FIR-FV2V scenarios are 8176, 2007, and 1136, respectively. It should be noted that the potential of FIR in reducing the number of road fatalities is not significantly increased compared with RGIR. Because 87.6% of the total driving mileage of vehicles is distributed on urban expressways, urban ordinary roads, expressways, Class I highways, and Class II highways covered by the RGIR scenario. Only 12.4% of the total driving mileage of vehicles is driven on Class III highways, Class IV highways, and substandard roads additionally covered by FIR. In the 12.4% of total driving mileages, AVs and V2V can also reduce traffic fatalities. Synergetic scenarios with RGIR can reduce more road fatalities in the early stage than scenarios with APIR. Faster coverage of IR can realize the collision avoidance potential earlier. For RGAV-RGIR-HV2V and RGAV-APIR-HV2V scenarios, the estimated annual road fatalities in 2030 are 37,834 and 41,505, respectively. In 2040, the estimated road fatalities in both scenarios are 10,013, because the IR has completely covered the selected road types in both scenarios.

The speed of decline is determined by the equipment rate of V2V. If IR coverage is high and V2V is only equipped to partially and fully AVs, road fatalities will decline slowly from 2025, and the high decline will not be realized until the proportion of partially and fully AVs is high enough. The fatalities are estimated to be 57,737, 44,720, 16,760, and 2943 in 2025, 2030, 2040, and 2050 in scenario RGAV-FIR-APV2V, which is similar to the declining trend of RGAV-RGIR-APV2V and SAV-FIR-APV2V. Compared to scenarios with APV2V, scenarios with HV2V and FV2V have a benefit space in the figure brought by a faster decline, although the estimated annual death toll in 2050 is close. V2V should be equipped as early as possible, with as many vehicle types as possible to reduce the fatalities related to road crashes more quickly.

### 3.3. Deployments for Achieving SDG Target 3.6 in 2030

The development of AV, IR, and V2V technology serves as an occasion to attain SDG target 3.6 to reduce road traffic fatalities and injuries by 50% by 2030. Compared with 2020, the baseline road fatalities are estimated to rise slightly to 63,972 in 2030, with a decrease of 36% in road fatalities per billion kilometers and an increase of 58% in vehicle stocks. It will be impossible to reduce the annual traffic fatalities by 50% by 2030 if new technologies are not introduced.

**Figure 4:**
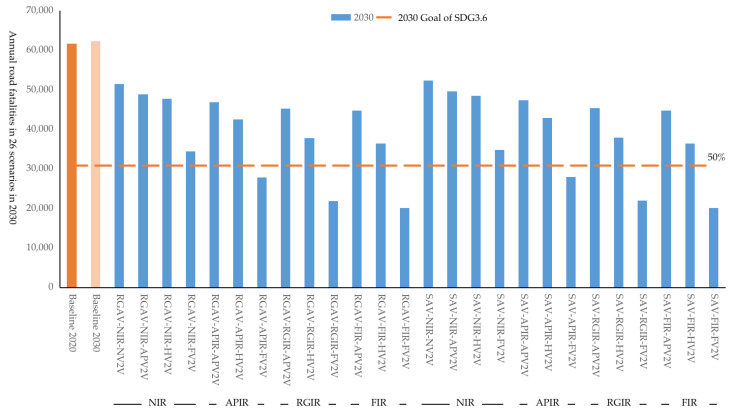
Estimated annual road fatalities in 26 scenarios in 2030 in China.

Corresponding ambitious SDG 3.6 goals can only be achieved through the synergetic development of autonomous vehicles, intelligent roads, and V2V. In 2030, 6 out of 26 synergetic scenarios meet the target for reducing injuries by 50% compared to 2020, including RGAV-APIR-FV2V, RGAV-RGIR-FV2V, RGAV-FIR-FV2V, SAV-APIR-FV2V, SAV-RGIR-FV2V, and SAV-FIR-FV2V, as shown in Figure 4. Deploying intelligent roads as planned (APIR) and fully equipped V2V (FV2V) are required for achieving the SDG 3.6 goal. There will be 27,793, 21,917, 20,143, 27,969, 21972, and 20,164 road traffic fatalities in the six scenarios in 2030, lower than 50% of the fatalities in 2020. In addition, there are 6 out of 26 scenarios that are close to achieving the goal in 2030, namely RGAV-NIR-FV2V, RGAV-RGIR-HV2V, RGAV-FIR-HV2V, SAV-NIR-FV2V, SAV-RGIR-HV2V, SAV-FIR-H2V. The estimated road fatalities in 2030 are 34,480, 37,834, 36,424, 34,774, 37,948, and 36,464, respectively. In general, to achieve the ambitious goal of halving traffic fatalities by 2030, it is necessary for all vehicles to be equipped with V2V, otherwise the possibility of achieving the 2030 goal will be lost. On the basis of FV2V, IR should at least cover the road type selected in the APIR scenario to achieve SDG target 3.6. FIR is unnecessary to achieve the 2030 goal because FIR needs to cover 4.67 times the mileage of RGIR and APIR, which will waste a lot of economic resources.

### 3.4. Cumulative Reductions in Road Fatalities

**Figure 5:**
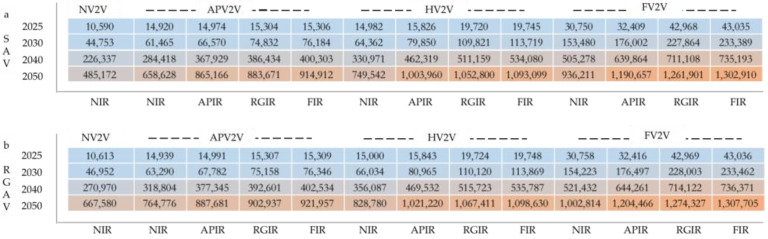
(**a**,**b**), Cumulative reductions in road fatalities for each AV synergetic scenarios: SAV (**a**), RGAV (**b**). Panel rows show variation by V2V equipment (scenarios NV2V, APV2V, HV2V, and FV2V, Table 1); columns show variation by IR scenarios (abbreviations as in Table 1).

The two scenarios with the largest cumulative reductions in fatalities are RGAV-FIR-FV2V and SAV-FIR-FV2V, which can reduce 1308 thousand and 1303 thousand road fatalities from 2015 to 2050. The two scenarios with the lowest cumulative fatalities avoided are RGAV-NIR-NV2V and SAV-NIR-NV2V: only 668 thousand and 485 thousand fatalities could be reduced cumulatively. The greater the investment in IR and V2V, the more cumulative road fatalities could be reduced. Comparing the results in Figure 5a,b, it can be observed that the cumulative fatalities reduced are similar to synergetic scenarios with the same V2V and IR (the same location in a and b). Especially in the synergetic scenarios with more positive deployment in IR and V2V, the difference in the cumulative reductions in fatalities brought by RGAV and SAV is smaller. RGAV-FIR-FV2V is estimated to only reduce 4795 more fatalities than SAV-FIR-FV2V cumulatively in 2015–2050. This reveals that when choosing to invest more in the development of IR and V2V, it is not necessary to invest too much in AV to realize the RGAV scenario, because the additional benefits brought by the RGAV in synergetic scenarios are not so high. However, when only AV is selected for development, without IR and V2V, it is important to attempt to realize RGAV. The 2015–2050 cumulative fatalities reduction in RGAV-NIR-NV2V is 182 thousand more than that in the SAV-NIR-NV2V scenario. Increasing V2V investment while reducing IR investment is a good way to achieve similar safety benefits. The 2015–2050 cumulative fatalities reduced by SAV-FIR-APV2V are 915 thousand, while 2015–2050 cumulative fatalities reduced by SAV-APIR-HV2V and SAV-NIR-FV2V are estimated to reduce 1004 thousand and 936 thousand fatalities.

### 3.5. Cumulative Savings of Crash-Related Economic Costs

**Figure 6:**
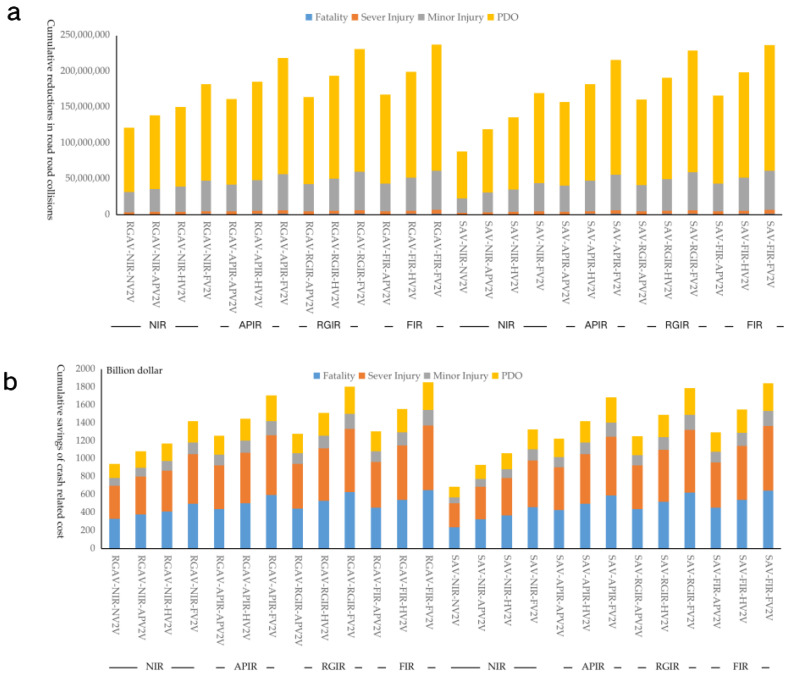
2015–2050 cumulative reductions in road injuries and savings in crash-related economic costs for 26 synergetic scenarios. (**a**) Cumulative reductions in road fatalities, serve injuries, minor injuries, and property damages only (PDO); (**b**) cumulative savings of crash-related economic costs.

The findings indicate that increasing the availability of AVs, IRs and V2V will have a large effect on preventing different kinds of road traffic collisions and reducing crash-related economic costs. The following results summarize the evaluated impact of 26 synergetic scenarios on collision avoidance and economic cost reduction in China. Of the 26 synergetic scenarios accessed, RGAV-FIR-FV2V would have the largest cumulative reductions in crashes and crash-related economic costs, resulting in 1.3 million fewer fatalities, 5.3 million fewer severe injuries, 54.9 million fewer minor injuries, 176.0 million fewer PDO collisions, and reducing crash-related costs by 1853 billion dollars cumulatively in 2015–2050 in China. These estimates are far ahead of the other synergetic scenarios, as shown in Figure 6. Together with development of AVs, IRs, and V2V, cumulative 2015–2050 road crash-related economic costs could be reduced by at least USD 1226 billion for the SAV-APIR-APV2V scenario. With both deployments of AVs and V2V, cumulative 2015–2050 road crash-related economic costs could be reduced by USD 934 to 1421 billion. Only increasing the availability of AVs, SAV and RGAV could save USD 688 billion and USD 947 billion in crash-related economic costs cumulatively from 2015 to 2050. The economic costs that could be reduced by AVs, IRs, and V2V are beyond imagination.

## 4. Discussion and Policy Implications

It is urgent to identify possible related policy options to determined the best choice for lowing road fatalities and injuries in China. The result shows that there would still be many road traffic fatalities and injuries in China if AVs, IRs, and V2Vs are not introduced to transportation in the future.

When formulating the development policy for AVs, the government should also simultaneously formulate the policy for IRs and V2V to maximize the safety benefits. A single technology alone cannot achieve a deep reduction in road crashes. Therefore, a series of technology and policy portfolios are needed for the road safety target. The evaluation of 26 synergetic scenarios suggests that the rapidly deployed autonomous vehicles (RGAV) can only bring 0.67, 2.72, 28.1, and 89.9 million cumulative 2015–2050 reductions in road fatalities, severe injuries, minor injuries, and PDO crashes, resulting in USD 946.2 million of cumulative 2015–2050 reduction in crash-related economic costs. In addition, RGAP with full equipment of V2V and full coverage of intelligent roads can result in 1.3 million fewer fatalities, 5.3 million fewer severe injuries, 55.0 million fewer minor injuries, 176.0 million fewer PDO collisions of the cumulative 2015–2050 reduction compared with the baseline scenario, resulting in reducing crash-related costs by USD 1854 billion cumulatively 2015–2050 in China.

AVs, IRs, and V2V play different roles in safety benefits. The foundation of the decline is the development of autonomous vehicles. The extent of safety benefits is largely affected by IR coverage. The more types of roads covered by intelligent roads, the more road crashes and related economic costs can be reduced in the future. If all road types are selected to be intelligent roads, the potential safety benefits will be great. However, the time to realize the maximum safety benefit depends on the equipment policy of V2V. V2V should be equipped with as many vehicle types as possible and as early as possible to reduce road crashes more quickly. SAV-FIR synergetic scenarios with different V2V sub-scenario would have a wide range of safety benefits on 2015–2050 cumulative reductions, ranging from 922 to 1308 thousand fewer road fatalities and a reduction of USD 1307 to USD 1854 billion crash-related economic costs.

In general, 6 out of 26 synergetic strategies are identified to achieve the ambitious SDG 3.6 goal of halving traffic fatalities by 2030. It is necessary that all vehicles should be equipped with V2V. The goal cannot be achieved in 2030 if only AV is deployed without the deployment of IR and V2V. On the basis of FV2V, IR should at least cover the road type selected in the APIR scenario to achieve the SDG 3.6 target. The SAV-APIR-FV2V is the easiest deployment plan to achieve the 2030 goal. From the perspective of China, it is not difficult to install V2V equipment in all vehicles before 2030 if the policy on mandatory installation of On-Board Unit (OBU) in all vehicles is in place. The price of an OBU in is approximately 1500 Chinese RMB (approximately USD 230). On the basis of APV2V, the government should formulate a series of policies to improve the penetration of V2V to a high enough level. For example, the government provides economic subsidies for vehicles equipped with OBU, or introduces financial policies to achieve zero cost to install OBU. Increasing V2V investment while reducing IR investment is a good way to achieve similar safety benefits.

Even if the full coverage of IR and full equipment of V2V are realized, RGAV-FIR-FV2V cannot avoid all road fatalities. There will still be 1136 deaths due to traffic accidents here in 2050. The passive safety technologies used to protect passengers after collision still require more attention and further development.

There are some important topics that require further research. The economic investment of deploying AVs, IRs, and V2V has not been evaluated. After the cost needs of each synergetic scenario are assessed, the safety benefit–cost analysis can be conducted to select the best economic policy to deploy the AVs, IRs, and V2V for road safety. The hardware cost of installing V2V on a vehicle is very low compared with the investment required for the hardware cost of an intelligent road per kilometer. Compared with full equipment of V2V, the deployment of a fully AV is much more expensive. Cost–benefit analysis is important for decision-makers to design strategies and policies on the deployment of AVs, IRs, and V2V, which is our ongoing research. Meanwhile, the safety impacts of 26 deployment scenarios are quantified and discussed in detail in this paper. Six synergetic scenarios that can meet the SDG 3.6 target are identified. However, the scenario analysis method is difficult to employ for identification of the best deployment solutions under a specific target. Based on the quantitative model in this paper, the deployment solutions selection model needs to be further developed to identify the optimal deployment solutions under a given safety target such as the SDG 3.6 target.

## 5. Conclusions

If AVs or IRs are not introduced to transportation, the estimated baseline road fatalities will rise slightly to 63,972 in 2030 and then decline slightly to 60,876 and 58,421 in 2040 and 2050, respectively. In 2030, 6 out of 26 synergetic scenarios meet the target for reducing injuries by 50% compared to 2020, including RGAV-APIR-FV2V, RGAV-RGIR-FV2V, RGAV-FIR-FV2V, SAV-APIR-FV2V, SAV-RGIR-FV2V, and SAV-FIR-FV2V. The estimated road fatalities are 27,793, 21,917, 20,143, 27,969, 21972, and 20,164 in the six scenarios in 2030, lower than 50% of the fatalities in 2020.

RGAV-FIR-FV2V would have the largest safety benefits, resulting in 1.3 million fewer fatalities, 5.3 million fewer severe injuries, 54.9 million fewer minor injuries, 176.0 million fewer PDO collisions, and reducing crash-related costs by USD 1853 billion cumulatively in 2015–2050 in China. SAV-FIR-FV2V would have the second largest safety benefits, resulting in 1.3 million fewer fatalities, 5.3 million fewer severe injuries, 54.8 million fewer minor injuries, 175.3 million fewer PDO collisions, and reducing crash-related costs by USD 1847 billion cumulatively in 2015–2050 in China.

With deploying IRs and V2V as planned, the deployment of SAV-APIR-APV2V can avoid 0.87 million fatalities, 3.53 million severe injuries, 36.36 million minor injuries and 116.43 million PDO collisions, resulting in a saving of USD 1226 billion of crash-related costs cumulatively in 2015–2050 in China. Only increasing the availability of AVs, SAV-NIR-NV2V and RGAV-NIR-NV2V could save 688 billion and 947 billion dollars of crash-related economic costs cumulatively from 2015 to 2050.

There are some strategies for decision-makers to design policies for deploying AVs, IRs and V2V. Compared with only deploying AVs, increasing the availability of IRs and V2V while reducing the deployment of fully AVs can achieve larger safety benefits in China. Increasing the deployment of V2V while reducing the deployment of IRs can achieve similar safety benefits sometimes. The deployment of AVs, IRs, and V2V plays different roles in achieving safety benefits. Increasing the availability of IRs could improve the upper limit of safety benefits. For the same deployment of IRs, increasing the deployment of V2V would speed up reaching the upper limit of safety benefits.

In general, the main contributions of the study are summarized as follows: (1) A bottom-up analytical framework was developed to evaluate the safety benefits from the deployment of AVs, IRs, and V2Vs in China from 2020 to 2050, which help us understand the variation of road injuries by the deployments of AVs, IRs and V2V; (2) Quantification of the safety benefits of avoiding road injuries and reducing crash related economic costs from the deployments of AVs, IRs, and V2Vs in China in 26 scenarios; (3) Clarification of different roles and contributions of deployment of AVs, IRs, and V2V in achieving safety benefits; (4) Identification of six synergetic scenarios that can meet the SDG 3.6 target for reducing casualties by 50% in 2030; (5) The framework developed in this study can be applied to evaluate the safety benefits from deployments of AVs, IRs, and V2V in other countries.

## Figures and Tables

**Table 1:** Description of the dimensions which are combined to generate the 48 scenarios.

Description of Scenarios	Description
Autonomous vehicle scenarios	AV market shares of total new vehicle sales
AV-No (NAV)	No AVs will be introduced in the market, including primary, partially, and fully AVs.
AV-Slow deployment (SAV)	Fully AVs are not allowed to enter the market due to policy barriers and immature technology, and only primary and partially AVs enter the market. The penetration rate of primary and partially AVs in new cars will be 40% and 10%, respectively in 2025, 35% and 55% in 2030, and 1% and 99% in 2050 [20].
AV-Rapid Growth deployment (RGAV)	Fully AVs are mature enough to be allowed to enter the market and will gradually become mainstream in the future. According to the roadmap (Ministry of Industry and Information Technology of China 2021), the penetration rate of primary, partially, and fully AVs in new cars reach 40%, 9%, and 1%, respectively in 2025, 35%, 35%, and 20% in 2030, 1%, 9% and 90% in 2050.
Intelligent road deployment scenarios	Intelligent road shares of different types of roads
IR-No (NIR)	No intelligent roads will be built.
IR-As Planned (APIR)	Urban expressways, urban ordinary roads, expressways, Class I highways, and Class II highways are the road types selected to be changed to intelligent roads, which will be fully covered in 2030, 2035, 2030, 2035, and 2040, respectively [21].
IR-Rapid Growth (RGIR)	The construction of intelligent roads offers the potential faster achievement of early full coverage of selected roads. Urban expressways, urban ordinary roads, expressways, Class I highways, and Class II highways will be fully covered in 2025.
IR-Full coverage (FIR)	All road types are selected to be changed to intelligent roads. The construction of intelligent roads is faster. Urban expressways, urban ordinary roads, expressways, Class I highways, and Class II highways will be fully covered in 2025. Class III, and Class IV highways and substandard roads will be fully covered by 2030.
Vehicle-to-Vehicle scenarios	Vehicle types equipped with V2V
V2V-No (NV2V)	V2V will not be equipped for vehicles.
V2V-As Planned (APV2V)	Only partially and fully AVs are equipped with V2V technology.
V2V-All AV (HV2V)	All AVs are equipped with V2V technology since 2025.
V2V-Full equipped (FV2V)	All vehicles are equipped with V2V technology since 2025, including traditional vehicles.

**Table 2:** Comprehensive collision avoidance effectiveness of different levels of autonomous vehicles driving on the traditional or intelligent road.

Comprehensive Collision Avoidance Effectiveness		Traditional Vehicle	Primary AV	Partially AV	Fully AV
Traditional road	Only Vehicle	0.0%	36.8%	55.6%	89.1%
	Vehicle with V2V working	26.7%	60.4%	74.7%	92.0%
Intelligent road	Vehicle on IR	26.7%	92.9%	94.6%	98.3%
	Vehicle on IR with V2V working	46.3%	95.2%	96.7%	98.8%

## Data Availability

Please contact the corresponding author if you have any question on the data.
